# Detection and cultivation of circulating tumor cells in gastric cancer

**DOI:** 10.1007/s10616-015-9866-9

**Published:** 2015-04-11

**Authors:** Katarina Kolostova, Rafal Matkowski, Robert Gürlich, Krzysztof Grabowski, Katarzyna Soter, Robert Lischke, Jan Schützner, Vladimir Bobek

**Affiliations:** 1 Department of Laboratory Genetics, University Hospital, Kralovske Vinohrady, Srobarova 50, 100 34 Prague, Czech Republic; 2 Division of Oncological Surgery, Department of Oncology, Wroclaw Medical University, Plac Hirszfelda 12, 53-413 Wrocław, Poland; 3 Lower Silesian Oncology Centre, Plac Hirszfelda 12, 53-413 Wrocław, Poland; 4 Department of Surgery, 3rd Faculty of Medicine, Charles University Prague and University Hospital Kralovske Vinohrady, Srobarova 50, Prague, Czech Republic; 5 Department of Gastrointestinal and General Surgery, Wroclaw Medical University, ul. M. Curie-Skłodowskiej 66, 50-369 Wrocław, Poland; 6 3rd Department of Surgery, First Faculty of Medicine, Charles University in Prague and University Hospital Motol, Prague, Czech Republic; 7 Department of Histology and Embryology, Wroclaw Medical University, Chalubinskiego 6a, 50-368 Wroclaw, Poland

**Keywords:** Gastric cancer, Circulating tumor cells, Metacell, CTC, Cultivation

## Abstract

Circulating tumor cells (CTCs) are important targets for treatment and critical surrogate markers when evaluating cancer prognosis and therapeutic response. A sensitive methodology for detecting CTCs in gastric cancer (GC) patients is needed. In this study we demonstrate a device for enrichment and cultivation of CTCs. In total, 22 patients with GC, all candidates for surgery, were enrolled in the study. Peripheral blood samples were collected before surgery, and patients were re-evaluated within operation and divided into two groups: resectable and non-resectable GC. A new size-based separation test for enrichment and cultivation of CTCs was used (MetaCell^®^). In addition to cytomorphological analysis, gene expression of tumor associated genes (Cytokeratin-18, Cytokeratin-19, Cytokeratin-20, Cytokeratin-7, EPCAM, MUC1, HER2, EGFR) and of leukocyte markers (e.g. CD45, CD68) was tested in enriched CTC fractions. CTCs were detected in 59 % of the patients studied (n = 13/22). CTCs were detected in seven patients of the resection group (7/10, 70 %) and six of the non-resectable group (6/12, 50 %). Enrichment of the viable CTCs allowed subsequent successful cultivation in vitro. The cytomorphological characterization of the CTCs was a prerequisite of random gene expression testing in CTC-positive samples. In CTC-positive samples gene expression of cytokeratin 18 and 19 was elevated in comparison to the whole blood gene expression analysis. CTCs were found to be present in both resectable and non-resectable gastric cancer patients. The size-based separation platform for CTCs may be used for in vitro cultivation, as well as in subsequent molecular analysis if desired. The sensitivity of CTC-detection could be enhanced by the combination of cytomorphological and molecular analysis.

**Electronic supplementary material:**

The online version of this article (doi:10.1007/s10616-015-9866-9) contains supplementary material, which is available to authorized users.

## Introduction

Metastatic dissemination is an important prognostic factor for patients with gastro-intestinal cancer. Exact staging is crucial to determine appropriate multimodal therapeutic strategies. The current staging method for gastric cancer (GC) is based on the staging system of the International Union against cancer Tumor-Node-Metastasis (TNM), in which the degree of tumor penetration (pT) and nodal status (pN) are the two main prognostic indicators. Early stage patients are considered for surgery. However, approximately 50 % of GC patients suffer from tumor relapses even after radical surgery (Marrelli et al. 2005).

Many research groups have focused on the identification of new potential biomarkers and novel tests, yet their specificity and sensitivity in a clinical setting frequently go reported. Recently, in advanced GC, measurement of HER2-expression is being recommended when selecting patients for treatment with Trastuzumab (Duffy et al. 2013). Circulating Tumor Cells (CTCs) and disseminated tumor cells (DTCs) could be rare events of primary tumor progression, which could be used for identification of cancer recurrence or progression risk. The methodology for CTC-detection in gastrointestinal cancer has been recently reviewed elsewhere (Kin et al. 2013). The development of new isolation platforms for CTCs is well supported by the need for new predictive markers in clinical treatment.

The real number of CTCs analyzed in peripheral blood (PB) in gastrointestinal cancer (colorectal cancer, GC, oesophageal cancer) is low compared with other malignancies such a breast and prostate cancer. The absolute (median) numbers in metastatic colorectal carcinoma (mCRC) are reported as 1–2 CTCs/7.5 mL of blood in mCRC, 3–5 CTCs/7.5 mL of blood in metastatic prostate cancer, and 6–7 CTCs/7.5 mL of blood in metastatic breast cancer (Negin and Cohen 2010; Hiraiwa et al. 2008; Moreno et al. 2001; Cristofanilli et al. 2012).

Follow-up studies in GC patients suggest that CTC-positive cases with an increased burden of CTCs were associated with a poorer prognosis than CTC-negative patients, and the situation was similar for DTCs (Wang et al. 2009). Both localized and metastatic GC can shed a detectable concentration of CTCs into the blood. The presence of CTCs in the circulation indicates a high risk of tumor recurrence as well as unfavourable clinical outcomes, even for early GC (Zhang and Ge 2013).

The prognostic use of CTCs in GC has been reported in several studies (Arigami et al. 2011; Saad et al. 2010; Pituch-Noworolska et al. 2007; Yeh et al. 1998; Koga et al. 2008; Illert et al. 2005; Uen et al. 2006). For GC, the presence of CTC and tumor markers (e.g. EpCAM/CK8/CK18/C19) seems prognostically the most relevant (Hiraiwa et al. 2008; Matsusaka et al. 2010). Based on the data analyzed, detection of CTCs may provide a useful non-invasive method for prognosis, as well as a means of confirming a GC diagnosis.

We have developed an easy and highly sensitive methodology for detecting CTCs in GC patients, namely the MetaCell^®^ platform. In this study we demonstrate its use for enrichment, separation and cultivation of CTCs.

## Materials and methods

### Patients

To date, 22 patients with diagnosed GC have been enrolled in the study. All patients had GC localized within 5 cm of the gastro-oesophageal junction, and were candidates for surgery. The patients’ details are shown in Table 1.

**Table 1:** Patients characteristics (22 patients in total, median age 68,75 years)

T stage	Patients (N)	CTC positive (N)	%
T1	3	1	33.3
T2	1	1	100
T3	8	5	62.5
T4	10	5	50
*N stage*			
N0	3	1	33.3
N1	8	3	37.5
N2	6	4	66.67
N3	5	4	80
*M stage*			
M0	17	10	58.8
M1	5	2	40
*Disease stage*			
I	3	1	33.3
II	4	2	50
III	10	7	70
IV	5	2	40

Peripheral blood was collected prior to surgery. For each patient approximately 8 mL of venous blood was drawn from the antecubital veins and placed into S-Monovette tubes (Sarstedt AG & Co., Nümbrecht, Germany) containing 1.6 mg EDTA/ml blood as an anticoagulant. The samples were processed at room temperature using an isolation procedure completed within 24 h of the blood draw.

The ethics committees of all participating universities and hospitals approved the study protocol according to the Declaration of Helsinki. All patients also provided written consent.

### CTC enrichment and culture

A new size-based separation method for viable CTC-enrichment from unclotted Peripheral blood (PB) was recently introduced (MetaCell^®^, MetaCell s.r.o., Ostrava, Czech Republic). The process is based on the filtration of PB through porous polycarbonate membrane (pores of 8 μm diameter, MetaCell s.r.o., Ostrava, Czech Republic). Successive blood transfer into the filtration tube in several steps is preferred, to prevent blood clotting on the membrane filter. The PB filter flow is supported naturally by capillary action of the absorbent material touching the membrane filter. Afterwards, the membrane filter, which is kept in a plastic ring, is transferred into the 6-well cultivation plate; RPMI medium (Sigma-Aldrich, Munich, Germany) is added to the filter top and CTCs are cultured on the membrane in vitro, under standard cancer cell culture conditions (37 °C and 5 % atmospheric CO_2_, ) and observed by inverted microscope. Alternatively, viable CTCs may be observed under a fluorescence microscope applying vital nuclear stain (NucBlue™, Life Technologies, Bleiswijk, Netherlands) and/or vital cytoplasmic stain (Celltracker™, Life Technologies, Bleiswijk, Netherlands). The CTCs are grown in an FBS (Sigma-Aldrich, Germany) enriched RPMI medium (10 %) for a minimum of 14 days and are analyzed by means of histochemistry (May-Grünwald staining (Bio-Optica, Milan, Italy) and immunohistochemistry using the tumor specific antibodies to determine the cell origin (mouse monoclonal anti-cytokeratin peptide 18-FITC antibody (Sigma), DAPI (Sigma), Prolong Gold Antifade Reagent (Life Technologies, Carlsbad, CA, USA).

Next, the enriched CTC fraction can be transferred from the membrane and cultured directly on a plastic surface or microscope slide. Microscope slide culturing is preferred if immunohistochemistry/immunofluorescence analysis is planned. If an intermediate CTCs-analysis is needed, the CTC-fraction is transferred in PBS (1.5 mL) to the cytospin slide. The slide is then dried for 24 h and analyzed by immunohistochemistry.

**Fig. 1:**
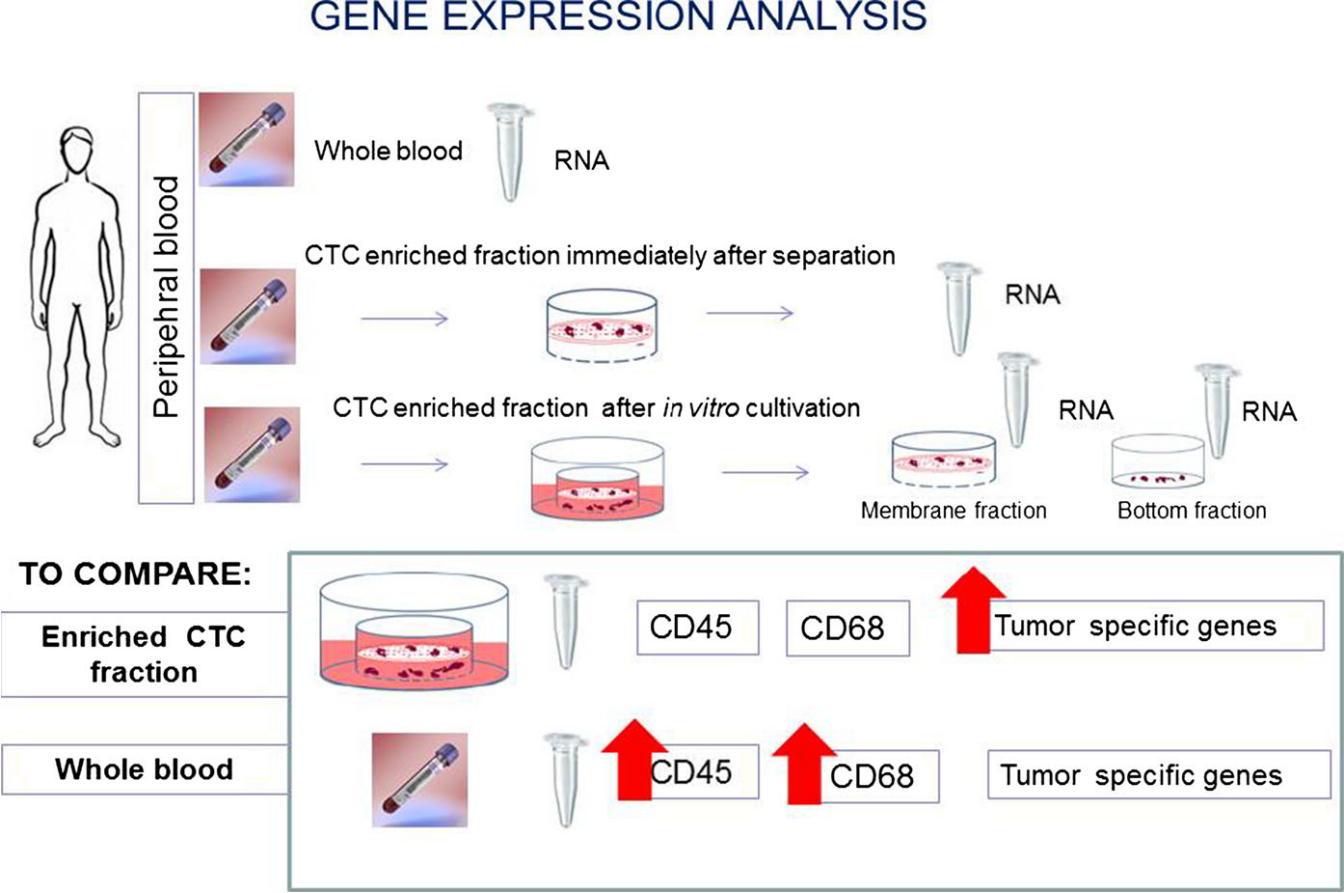
Gene expression analysis of the enriched CTC-fractions

Additionally, to confirm the origin of the cells on the separation membrane, the CTC-gene expression analysis was performed in parallel with the immunohistochemistry. The cell fraction enriched on the membrane without in vitro culturing was assigned as “virgin” CTCs. The reported gene expression analysis was performed for virgin CTCs (see Fig. 1). For proceeding with an in vitro culture of these cells, we analyzed the gene expression of the cells captured (or grown) on the membrane (so called membrane fraction) as well as the fraction of cells that were able to overgrow the membrane and set up a new cell culture on the bottom of the cultivation plastic (see Fig. 1, “bottom fraction”). The gene expression of the tumor-associated markers in the CTC-enriched fractions was then compared with the gene expression of these markers in the whole blood RNA.

### Cytomorphological analysis

Stained membranes were examined using light microscopy in two steps: (1) screening at ×20 magnification to locate cells; (2) observation at ×40–×60 magnification for detailed cytomorphological analysis. Isolated cells and/or clusters of cells of interest (whether immunostained or not) were selected, digitized, and examined by an experienced researcher and/or pathologist. CTCs were defined as cells presenting all the following criteria: (1) nuclear size ≥10 μm); (2) irregular nuclear contour; (3) presence of visible cytoplasm; (4) high nucleus-to-cytoplasm ratio; (5) prominent nucleoli; (6) proliferation activity; (7) formation of 3D cell layers.

### Gene expression analysis

For GC patients where CTC-presence was confirmed cytomorphologically (n = 10), gene expression analysis was subsequently performed. Gene expression analysis is best done in parallel with immunohistochemistry, in order to provide evidence of the epithelial origin of the captured CTCs. Gene expression of tumor associated genes (Cytokeratin-18, Cytokeratin-19, Cytokeratin-20, Cytokeratin-7, EPCAM, MUC1, HER2, EGFR) was tested, as was that of leukocyte markers (e.g. CD45, CD68), with endogenous control provided by the beta-actin gene. The gene expression of the CTCs captured on the membrane is compared to the gene expression of the tumor markers in the whole blood and between the “membrane fraction” and “bottom fraction” (see Fig. 1). The cells on the membrane were lysed by RLT-buffer with β-mercaptoethanol (Qiagen—CEEMED, Praha, Czech Republic), RNA was then isolated using RNeasy Mini Kit (Qiagen). The RNA from whole blood was isolated with a modified protocol. The protocol is including the erythrocyte-lysing step. The quality/concentration of RNA was measured by NanoDrop (ThermoScientific). As there are only a few hundred cells on the membrane, the median concentration of RNA was quite low (5–10 ng/μl). For cDNA production we used the High Capacity cDNA Reverse Transcription Kit (Life Technologies). For Gene expression analysis we employed Taqman chemistry including Taqman MGB—probes for all the above-mentioned genes (Life Technologies). (A list of Taqman probes we employed is given in the supplementary material.) The gene expression results report the CTC positivity in case of an increased gene expression of tumor-associated genes in CTC-fractions in comparison to the whole blood RNA.

## Results

**Fig. 2:**
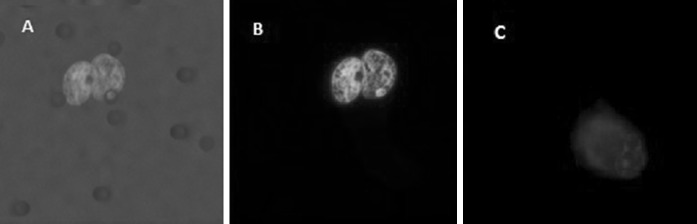
**a** CTC with two nuclei captured and cultured on a membrane filter with visualized filter pores. **b** Nucleus counterstained with DAPI. **c** CTC captured and cultured on a membrane filter, incubated with CK-18-FITC antibody with an unspecifically visualized micronucleus, with nucleus of irregular shape counterstained with DAPI

**Fig. 3:**
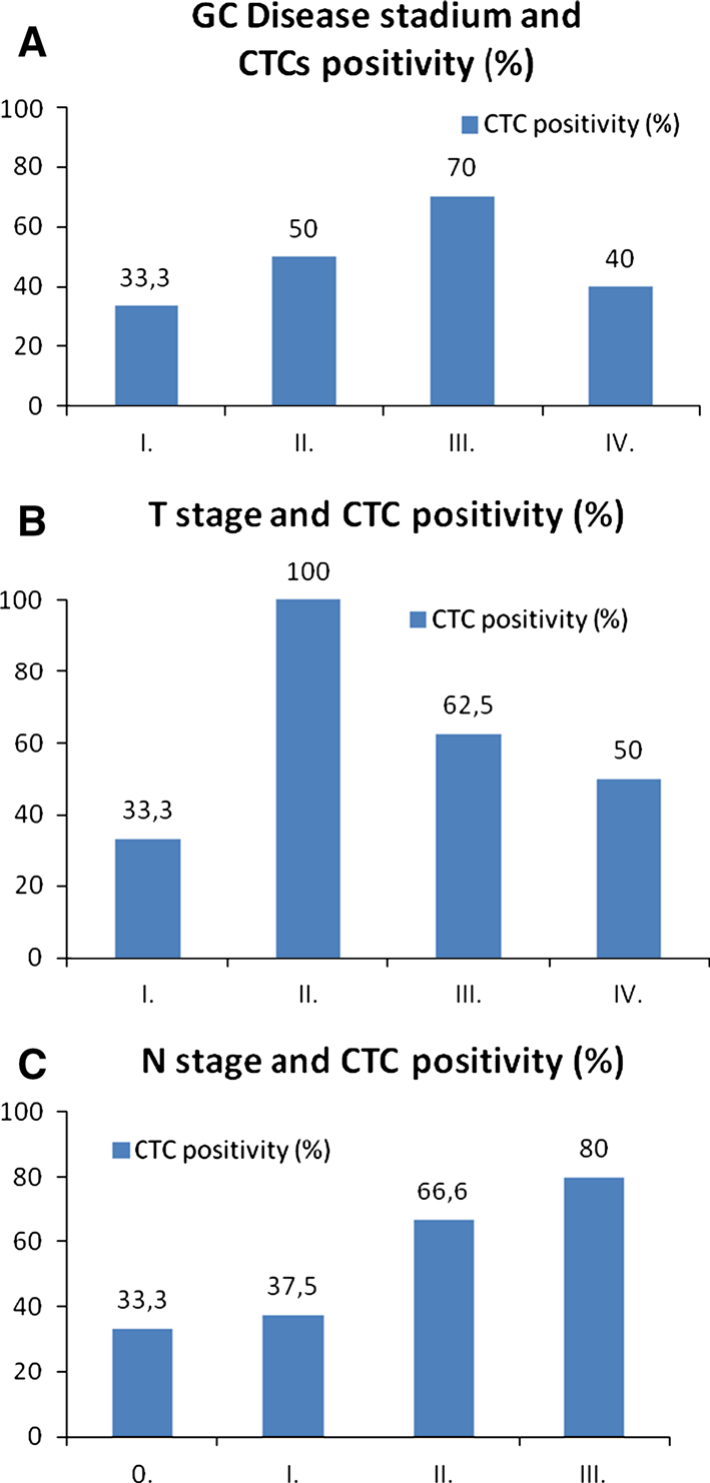
**a** Gastric cancer disease stage and CTC–positivity ratio. **b** T stage in Gastric cancer and CTC positivity. **c** N stage in Gastric cancer and CTC positivity

**Fig. 4:**
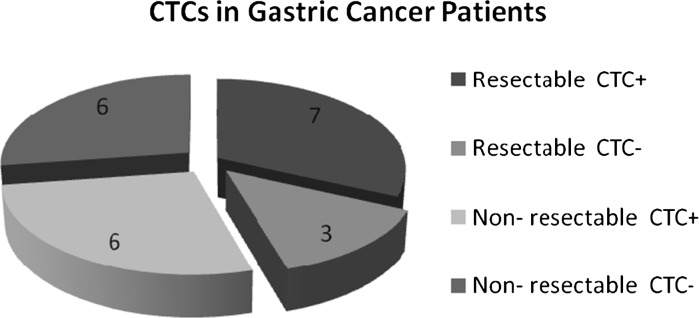
Resectability of gastric cancer and CTCs positivity

We report successful CTC isolation in 59 % of GC patients (n = 13/22). The CTC cell morphology and immunohistochemistry is shown in Fig. 2. The captured cells were stained positively for CK18. The CK18 molecule has generally been accepted as a marker of cancer with an epithelial origin (Fareed et al. 2012). A summary of the CTC positivity statistics is given in Table 1; Figs. 3 and 4. Overall, the size-based filtration approach enabled the capture of viable CTCs. We proved the viability of the CTCs by culturing the CTC cells in vitro, and confirming with further analysis (e.g. immunohistochemical or molecular). If desired the DNA and RNA molecules may be used for further mutational and gene expression testing. We do not have a recent survival analysis of the group of patients tested, but thanks to the data obtained, we are able characterize CTC dissemination in the pre-defined patient sub-groups based on disease stage (see Fig. 3b, c). It is of interest that 70 % of patients with resectable GC were CTC-positive, while patients deemed unresectable were only 50 % positive. The CTC-positivity rates in resectable/non-resectable GC patients and other GC-subgroups were compared by Chi square testing. No significant difference was found comparing resectable and non-resectable group of patients in our study (*P* = 0.0623).

Although the study group of GC patients was relatively small, the CTC-positive rates correlate with the disease stage as well as lymph node involvement. Samples from GC patients where CTC-presence was proven cytomorphologically were eligible for subsequent gene expression analysis. The gene expression results can serve as additional evidence of epithelial cell-origin alongside the immunohistochemistry. Analyzing the molecular character of the CTCs we may conclude that in our GC CTCs samples, an increased cytokeratin-18 and cytokeratin-19 expression was seen in all of the tested samples, with some redundant EpCAM and MUC1 expression as well. In none of the tested samples was cytokeratin-7 found. Gastric tumor histology confirmed the expression of HER2 and EGFR. In all the samples tested CD45 and CD68 expression was reported, but the expression level was much lower in the enriched CTC-samples on the membrane or after in vitro culture. We can report that the mRNA transcripts of cytokeratins and other possible epithelial markers are more abundant in the enriched CTC-fractions than in the whole blood. These facts combined with the cytomorphological analysis seem to be reasonable evidence for CTC-presence and successful CTC-detection. A combination of vital cell stain and subsequent gene expression analysis of the stained cells on the membrane could give an immediate answer as to what kind of the cells have been isolated.

## Discussion

The detection of malignant cells in blood has been established for many years (Engell 1955). More recent studies have demonstrated the malignant nature of CTCs (Fehm et al. 2002). In the early days of CTC research several groups attempted to identify and detect CTCs in PB but only by reverse transcription polymerase chain reaction (RT-PCR). In their study Soeth et al. (1997) noted that GC patients found positive for CK 20 mRNA (17 % of 30 patients) had significantly shorter survival times than those who tested negative. Miyazono et al. (2001) examined the presence of CTCs in blood samples from 57 GC patients. After density gradient separation of CTCs, CEA-specific real-time RT-PCR was performed and correlated with the time course during the surgical procedure and the onset/advent of hepatic tumor recurrence. Interestingly, the authors were able to show that CEA-mRNA could not be detected in a control group of healthy volunteers or in 15 patients with benign disease. In contrast, a total of 21 GC patients (36.8 %) were positive for CTCs as detected by CEA-specific RT-PCR and positive rates correlated with depth of tumor invasion. In addition, the authors found that GC patients with high levels of CEA were more likely to develop systemic disease.

In our study we saw higher CTC-positive rates than previously reported. But this may be caused by the enrolling in our study of patients with more advanced stages of GC. Another possibility is that the combination of cytomorphological and molecular analysis could be more sensitive for CTC detection in GC cases than previously reported methods. The big advantage of our approach is the ability to obtain biological material, namely CTCs cells, which are suitable for further downstream molecular analysis, e.g. gene expression profiling.

With the introduction of immunomagnetic separation techniques, not only detection but also quantification of CTCs becomes possible (Allard et al. 2004). Matsusaka et al. (2010) showed that the number of CTCs before and during treatment is an independent prognostic and predictive marker in GC patients. Patients with more than 4 CTCs identified at 2 and 4-weeks after start of chemotherapy had a shorter median progression-free survival (PFS), (1.4, 1.4 months, respectively) than those with <4 CTCs (4.9, 5.0 months, respectively). Patients with more than 4 CTCs at 2 and 4-weeks after initialization of chemotherapy had shorter median overall survival (OS), (3.5, 4.0 months, respectively) than those with <4 CTCs (11.7, 11.4 months, respectively).This study was performed using immunomagnetic platform with magnetic beads labelled with antibodies to GC cells that express specific cells surface antigens (EpCAM, CK8, CK18, CK19 and CD45). All antibody-based enrichment techniques may be limited by the possible loss of cells without the expression of epithelial antigens as metastatic cells undergo epithelial-mesenchymal transition (EMT). Changes in the cytoskeleton of epithelial cells such as the regression of cytokeratin can be observed (Sun et al. 2011). Several size-based filtration devices seek to overcome this limitation of lower sensitivity of antibody methods (Zheng et al. 2010). Similarly, we have shown a successful filtration-based approach for CTC detection, finding that 59 % of GC patients in the study were CTC positive.

Uenosono et al. (2013) published a study covering 251 patients with resectable and non-resectable GC (Uenosono et al. 2013). CTCs were detected in 16 patients (10.8 %) of the resection group and 62 patients (60.2 %) of the non-resectable group. The OS rate for the entire cohort was significantly lower in patients with CTCs than in those without them (*P*  <  0.0001). A significantly smaller number of patients took part in our study, but a very high proportion of our “resectable” group were CTC positive (70 %). The significant difference between Uenosono and colleague’s study and ours was the CTC-detection platform. Uenosono employed an epithelial marker-dependent methodology (CellSearch^®^), whereas we used an antibody-independent technology platform (MetaCell^®^) that identifies CTCs by size. The choice of detection platform is important because of Epitelial-Mesenchymal Transduction (EMT), a widely reported prerequisite for metastasis, which may lead to underestimation of CTC numbers. The inadequacy of the EpCAM-based immunomagnetic capture method compared with the size-based filtration method has been reported in several studies for different cancers (Farace et al. 2011; Krebs et al. 2012).

The advantage of the filtration method that we used is not only a higher detection rate but the ability to separate viable cancer cells. Enriched viable CTCs can be cultured for downstream testing as shown by gene-expression analysis or for single-cell analysis to detect CTC heterogeneity. For the first time in the study of GC the reported platform enables separation of viable CTCs and their subsequent cultivation.

## Electronic supplementary material

Below is the link to the electronic supplementary material.

Supplementary material 1 (XLS 24 kb)

## References

[CR2] Allard WC, Matera J, Miller MC, Repollet M, Connelly MC, Rao C, Tibbe AG, Uhr JW, Terstappen LW (2004). Tumor cells circulate in the peripheral blood of all major carcinomas but not in healthy subjects or patients with nonmalignant diseases. Clin Cancer Res.

[CR3] Arigami T, Uenosono Y, Hirata M, Yanagita S, Ishigami S, Natsugoe S (2011) B7-H3 expression in gastric cancer: a novel molecular blood marker for detecting circulating tumor cells. Cancer Sci 102:1019–1024 10.1111/j.1349-7006.2011.01877.x 21251161

[CR4] Cristofanilli (2012). Circulating tumor cells (CTCs) in breast cancer: enumeration molecular analysis and targeting of metastatic disease.

[CR5] Duffy MJ, Lamerz R, Haglund C, Nicolini A, Kalousová M, Holubec L, Sturgeon C (2013). Tumor markers in colorectal cancer, gastric cancer and gastrointestinal stromal cancers: European group on tumor markers 2014 guidelines update. Int J Cancer.

[CR6] Engell HC (1955). Cancer cells in the circulating blood; a clinical study on the occurrence of cancer cells in the peripheral blood and in venous blood draining the tumour area at operation. Ugeskr Laeg.

[CR7] Farace F, Massard C, Vimond N, Drusch F, Jacques N, Billiot F, Laplanche A, Chauchereau A, Lacroix L, Planchard D, Le Moulec S, André F, Fizazi K, Soria JC, Vielh P (2011). A direct comparison of Cell Search and ISET for circulating tumour-cell detection in patients with metastatic carcinomas. Br J Cancer.

[CR8] Fareed KR, Soomro IN, Hameed K, Arora A, Lobo DN, Parsons SL, Madhusudan S (2012). Caspase-cleaved cytokeratin-18 and tumour regression in gastro-oesophageal adenocarcinomas treated with neoadjuvant chemotherapy. World J Gastroenterol.

[CR9] Fehm T, Sagalowsky A, Clifford E (2002). Cytogenetic evidence that circulating epithelial cells in patients with carcinoma are malignant. Clin Cancer Res.

[CR10] Hiraiwa K, Takeuchi H, Hasegawa H, Saikawa Y, Suda K, Ando T, Kumagai K, Irino T, Yoshikawa T, Matsuda S, Kitajima M, Kitagawa Y (2008) Clinical significance of circulating tumor cells in blood from patients with gastrointestinal cancers. Ann Surg Oncol 15:39092–39100 10.1245/s10434-008-0122-9 18766405

[CR11] Illert B, Fein M, Otto C, Cording F, Stehle D, Thiede A, Timmermann W (2005) Disseminated tumor cells in the blood of patients with gastric cancer are an independent predictive marker of poor prognosis. Scand J Gastroenterol 40:843–849 10.1080/00365520510015557 16109661

[CR12] Kin C, Kidess E, Poultsides GA, Visser BC, Jeffrey SS (2013). Colorectal cancer diagnostics: biomarkers, cell-free DNA, circulating tumor cells and defining heterogeneous populations by single-cell analysis. Expert Rev Mol Diagn.

[CR13] Koga T, Tokunaga E, Sumiyoshi Y, Oki E, Oda S, Takahashi I, Kakeji Y, Baba H, Maehara Y (2008) Detection of circulating gastric cancer cells in peripheral blood using real time quantitative RT-PCR. Hepatogastroenterology 55:1131–1135 18705345

[CR14] Krebs MG, Hou JM, Sloane R, Lancashire L, Priest L, Nonaka D, Ward TH, Backen A, Clack G, Hughes A, Ranson M, Blackhall FH, Dive C (2012). Analysis of circulating tumor cells in patients with non-small cell lung cancer using epithelial marker-dependent and-independent approaches. J Thorac Oncol.

[CR15] Marrelli D, De Stefano A, de Manzoni G (2005). Prediction of recurrence after radical surgery for gastric cancer: a scoring system obtained from a prospective multicenter study. Ann Surg.

[CR16] Matsusaka S, Chìn K, Ogura M, Suenaga M, Shinozaki E, Mishima Y, Terui Y, Mizunuma N, Hatake K (2010). Circulating tumor cells as a surrogate marker for determining response to chemotherapy in patients with advanced gastric cancer. Cancer Sci.

[CR17] Miyazono F, Natsugoe S, Takao S (2001). Surgical maneuvers enhance molecular detection of circulating tumor cells during gastric cancer surgery. Ann Surg.

[CR18] Moreno JG, O'Hara SM, Gross S, Doyle G, Fritsche H, Gomella LG, Terstappen LW (2001). Changes in circulating carcinoma cells in patients with metastatic prostate cancer correlate with disease status. Urology.

[CR19] Negin BP, Cohen SJ (2010). Circulating tumor cells in colorectal cancer: past, present, and future challenges. Curr Trea. Options Oncol.

[CR1] Pituch-Noworolska A, Kolodziejczyk P, Kulig J, Drabik G, Szczepanik A, Czupryna A, Popiela T, Zembala M (2007). Circulating tumour cells and survival of patients with gastric cancer. Anticancer Res.

[CR20] Saad AA, Awed NM, Abd Elkerim NN, El-Shennawy D, Alfons MA, Elserafy ME, Darwish YW, Barakat EM, Ezz-Elarab SS (2010) Prognostic significance of E-cadherin expression and peripheral blood micrometastasis in gastric carcinoma patients. Ann Surg Oncol 17:3059–3067 10.1245/s10434-010-1151-8 20563657

[CR21] Soeth E, Vogel I, Roeder C, Juhl H, Marxsen J, Krüger U, Henne-Bruns D, Kremer B, Kalthoff H (1997). Comparative analysis of bone marrow and venous blood isolated from gastrointestinal cancer patients for the detection of disseminated tumor cells using reverse transcription PCR. Cancer Res.

[CR22] Sun YF, Yang XR, Zhou J, Qiu SJ, Fan J, Xu Y (2011). Circulating tumor cells: advances in detection methods, biological issues, and clinical relevance. J Cancer Res Clin Oncol.

[CR23] Uen YH, Lin SR, Wu CH, Hsieh JS, Lu CY, Yu FJ, Huang TJ, Wang JY (2006). Clinical significance of MUC1 and c-Met RT-PCR detection of circulating tumor cells in patients with gastric carcinoma. Clin Chim Acta.

[CR24] Uenosono Y, Arigami T, Kozono T, Yanagita S, Hagihara T, Haraguchi N, Matsushita D, Hirata M, Arima H, Funasako Y, Kijima Y, Nakajo A, Okumura H, Ishigami S, Hokita S, Ueno S, Natsugoe S (2013). Clinical significance of circulating tumor cells in peripheral blood from patients with gastric cancer. Cancer.

[CR25] Wang GY, Li Y, Yu YM, Yu B, Zhang ZY, Liu Y, Wang SJ (2009). Detection of disseminated tumor cells in bone marrow of gastric cancer using magnetic activated cell sorting and fluorescent activated cell sorting. J Gastroenterol Hepatol.

[CR26] Yeh KH, Chen YC, Yeh SH, Chen CP, Lin JT, Cheng AL (1998) Detection of circulating cancer cells by nested reverse transcription-polymerase chain reaction of cytokeratin-19 (K19)–possible clinical significance in advanced gastric cancer. Anticancer Res 18:1283–1286 9615802

[CR27] Zhang ZY, Ge HY (2013) Micrometastasis in gastric cancer. Cancer Lett 336:34–45. doi:10.1016/j.canlet.2013.04.021 10.1016/j.canlet.2013.04.021 23624301

[CR28] Zheng S, Lin HK, Lu B, Williams A, Datar R, Cote RJ, Tai YC (2010). 3D microfilter device for viable circulating tumor cell (CTC) enrichment from blood. Biomed Microdevices.

